# Invasive pulmonary aspergillosis in patients with chronic obstructive pulmonary disease

**DOI:** 10.1186/2049-6958-8-59

**Published:** 2013-09-04

**Authors:** Nuri Tutar, Gokhan Metan, Ayşe Nedret Koç, Insu Yilmaz, Ilkay Bozkurt, Zuhal Ozer Simsek, Hakan Buyukoglan, Asiye Kanbay, Fatma Sema Oymak, Inci Gulmez, Ramazan Demir

**Affiliations:** 1 Department of Pulmonary Medicine, Erciyes University Faculty of Medicine, Kayseri 38039, Turkey; 2 Department of Infectious Diseases and Clinical Microbiology, Erciyes University Faculty of Medicine, Kayseri, Turkey; 3 Department of Microbiology, Kayseri, Turkey

**Keywords:** Aspergillosis, Chronic obstructive pulmonary disease, COPD, Invasive pulmonary aspergillosis

## Abstract

**Background:**

Invasive pulmonary aspergillosis (IPA) is an infection often occurring in neutropenic patients and has high mortality rates. In recent years, it has been reported that the incidence of IPA has also increased in patients with chronic obstructive pulmonary disease (COPD). The purpose of this study is to investigate the clinical and demographic characteristics and treatment responses of IPA in patients with COPD.

**Methods:**

Seventy-one patients with a positive culture of *Aspergillus* from lower respiratory tract samples were examined retrospectively. Eleven (15.4%) of these patients, affected with grade 3 or 4 COPD, had IPA.

**Results:**

*Aspergillus* hyphae were detected in lung biopsy in three (27.3%) out of 11 patients and defined as proven IPA; a pathological sample was not taken in the other eight (72.7%) patients, and these were defined as probable IPA. *Aspergillus* isolates were identified as six cases of *Aspergillusfumigatus* and three of *Aspergillusniger* in nine patients, while two isolates were not identified at species level. While five patients required intensive care unit admission, four of them received mechanical ventilation. The most common finding on chest X-ray and computed tomography (CT) (respectively 63.6%, 72.7%) was infiltration. Amphotericin B was the initial drug of choice in all patients and five patients were discharged with oral voriconazole after amphotericin B therapy. Six patients (54.5%) died before treatment was completed.

**Conclusions:**

IPA should be taken into account in the differential diagnosis particularly in patients with severe and very severe COPD presenting with dyspnea exacerbation, poor clinical status, and a new pulmonary infiltrate under treatment with broad-spectrum antibiotics and steroids.

## Background

Invasive Pulmonary Aspergillosis (IPA) is frequently seen in patients with hematologic malignancy, allogenic bone marrow transplantation, the late stage of HIV infection, solid organ transplantation, and chronic granulomatosis [1-3]. In addition, the number of studies stating that IPA is increasing in cases with chronic obstructive pulmonary disease (COPD) has gradually risen in recent years [3-7].

In COPD patients ciliary activity impairment, immunosuppression due to alveolar macrophage and neutrophil inhibition in those treated with steroids and also receiving broad-spectrum antibiotics, play a role in the development of IPA [8,9]. In a review, in which 1,941 patients followed up with the diagnosis of IPA were examined, COPD was detected in 1.3% of all cases [10]. In a study carried out in the years following this review, it was found that the frequency of IPA in COPD cases in 2007 had increased approximately more than twice with respect to the year 2000 in the same hospital [6]. This finding shows that the rate of IPA incidence in COPD patients is ever increasing. Based on this study carried out in Spain we planned to report our IPA cases with COPD. Hence, in the present study we aimed to investigate the clinical and demographic features of IPA in patients with COPD and also to examine the treatment outcomes and mortality rates.

## Methods

### Study population and case descriptions

This retrospective study was carried out on patients who applied to Erciyes University Hospital, which has 1,300 in-patient bed availability. It was approved by the Ethics Review Board of Erciyes University (2013/269).

COPD diagnosis was established according to the Global Initiative for Chronic Obstructive Lung Disease (GOLD) criteria [11]. In patients with COPD, Bulpa criteria were used for IPA diagnosis [5]. Accordingly, the diagnosis of “proven IPA” was established by means of the presence of hyphae compatible with *Aspergillus* in the examination of specimens taken from a pulmonary lesion through pulmonary biopsy or needle aspiration, and by the isolation of *Aspergillus* species in culture from any upper respiratory tract (URT) sample together with evidence of associated tissue damage. The diagnosis of “probable IPA”, it was established by the coexistence of host factors [patient with severe COPD according to GOLD (stage III or IV), usually treated with steroids, with recent exacerbation of dyspnoea, and suggestive chest imaging] and microbiological factors [*Aspergillus* isolation in LRT sample or two sequential positive serum galactomannan (GM) tests]. The diagnosis of “possible IPA” required host factors, but without microbiological proof, while “colonization” meant the patient was asymptomatic in spite of the isolation of *Aspergillus* in the lower respiratory tract samples.

In our study, the response to antifungal treatment was evaluated based upon the criteria determined by the European Organization for the Research and Treatment of Cancer (EORTC) and the Mycoses Study Group (MSG) [12]. Accordingly, a reduction of at least 25% in the diameter of radiological lesions, together with recovery in the findings of disease and disease-specific symptoms and the patient’s being alive at the end of treatment were defined as treatment success. As for treatment failure, it was defined as follows: radiological stabilization (the 0-25% resolution of lesion diameter) together with a slight or no recovery in the findings of disease and coexistent symptoms despite the fact that the patient survived; the occurrence of disease in new areas together with the worsening of findings and symptoms of disease clinically; radiological deterioration of pre-existing lesion; continuous isolation of fungus species from the infected area; the patient’s death within the 12 week-follow up period for whatever reason.

### Patient inclusion criteria and data collection

In the present study we examined the data on the isolation of *Aspergillus* from LRT samples in our microbiology laboratory which were sent from the chest disease or infectious disease clinics between January 2006 and December 2011. The inclusion criteria were as follows: *1-* the isolation of *Aspergillus* from LRT culture; *2-* patients had COPD in GOLD stage 3 (severe) or 4 (very severe); *3-* patients had a history of steroid use (systemic or inhaled) in the last 3 weeks before admission or had a history of antibiotic use in the last 3 months before admission; *4-* the isolation was not considered as colonization. The patients were diagnosed as “proven IPA” and “probable IPA”. There was no case of “possible IPA” since only patients with a positive culture for *Aspergillus* were included in the study. The files, discharge reports and x-rays of all patients, which were registered in the hospital data processing system, were checked. Accordingly, we registered: demographic features, history of smoking, presence of other coexisting diseases, pulmonary function test results, history of drug use, clinical symptoms, results of laboratory tests, imaging findings, hospitalization periods, intensive care unit (ICU) admission, invasive mechanical ventilation support , and response to treatment.

### Microbiological procedures

In the course of the study period, following the direct microscopic examination, all LRT specimens were cultured on Sabouraud dextrose agar (SDA) media with and without antibiotics. One of these was incubated at 37°C and the others in incubators at 25°C for 3 weeks and were evaluated as to whether isolation occurs 3 times in a week, or not. The identification of a colony presenting mould growth was carried out at species level according to macroscopic and microscopic morphology [13].

The galactomannan (GM) antigen level in serum was detected in all patients by Platelia *Aspergillus* Galactomannan EIA kits (Bio-Rad, Marnes-La-Coquette, France). The ratio of ≥ 0.5 ng/mL for GM was considered positive. A 1,3-beta D-Glucan level was detected by Fungitell kits (Associates of Cape Cod, East Falmouth, MA, USA) and a ratio of > 80 pg/mL was considered positive [2].

### Statistical method

The frequency, percentage, mean, standard deviation, median and interquartile range values from descriptive statistics are presented. The SPSS 15.0 packaged software was used for the analysis of data.

## Results

### Diagnostic and demographic characteristics of patients

In 71 patients *Aspergillus* had been isolatedin LRT samples. Eleven out of them (15.4%) had stage 3 or 4 COPD and these were the cases that applied to our hospital with complaints of dyspnea. As a result of lung biopsy in three cases (27.3%) out of these 11 patients, hyphae compatible with *Aspergillus* were seen and identified as proven IPA; in the other eight cases (72.7%), pathological specimens were not taken and these were identified as probable IPA.

Six *Aspergillus* strains were defined as *Aspergillus fumigatus* and three as *Aspergillus niger,* while identification at species level in the other two strains was not made.

The mean age was 65.1 ± 9.7 years and 9 patients (81.8%) were male. History of smoking was present in 8 cases (72.7%). Ten patients (90.9%) had been using a systemic steroid and 8 (72.7%) an inhaler steroid over the last 3 weeks. History of antibiotic use was present in four patients over the last 3 months. The average diagnosis time was 13.8 ± 3.8 days after admission and the average hospitalization period was 24.7 ± 13.9 days. Five cases had ICU admission and four of them received invasive mechanical ventilation (Table 1).

**Table 1:** **Clinical and demographic characteristics of 11 COPD patients with IPA**^a^

**Characteristics**	
Male	9 (81.8)
Female	2 (18.2)
Mean age, years	65.1 ± 9.7
Smoking history	
Yes	8 (72.7)
>40, pack years	4 (36.3)
Underlying disease	
Diabetes mellitus	1 (9.0)
Cardiovascular disease	3 (27.2)
Steroid treatment^b^	
Inhaled	8 (72.7)
Systemic use	10 (90.9)
Recent use of antibiotics^c^	4 (36.3)
Time to diagnosis, days	13.8 ± 3.8
Hospitalization period, days	24.7 ± 13.9
ICU admission	5 (45.4)
Mechanical ventilation	4 (36.3)
Death	6 (54.5)

^a^Data are presented as number (%) or average ± standard deviation.

^b^Within three weeks before applying, ^c^Within three months before applying.

### Symptoms, laboratory results and radiological findings

The most frequent symptoms were dyspnea (n = 11, 100%) and cough (n = 10, 90.9%). Sputum, fever and hemoptysis were other recorded symptoms (Table 2). Moreover, GM was positive in nine patients (81.8%). The median value for GM was 0.54 ng/mL. Beta D-Glucan was examined in five patients and was positive in three (60%) of them.

**Table 2:** Symptoms, laboratory and radiological findings of 11 COPD patients with IPA

**Symptom, n (%)**	
Fever	5 (45.4)
Cough	10 (90.9)
Dyspnea	11 (100)
Sputum	7 (63.6)
Hemoptysis	2 (18.1)
Laboratory, average ± SD or median (IQR)	
White blood cell, 10^3^/mL	13.5 ± 5.2
Hemoglobin, g/dL	12.0 ± 2.8
Thrombocyte, 10^3^/mL	352.1 ± 175.1
Sedimentation, mm/hour	88 ± 25
CRP, mg/L	117 ± 50
Galactomannan, ng/mL	0.54 (0.17-9.16)
Radiological findings, n (%)	
Chest X-ray	
Infiltration	7 (63.6)
Cavity	3 (27.2)
Nodule	3 (27.2)
Pleural fluid	2 (18.1)
Consolidation	2 (18.1)
Worsening radiological findings^a^	7 (63.6)
Computed tomography	
Infiltration	8 (72.7)
Nodule	4 (36.3)
Consolidation	3 (27.2)
Cavitation	3 (27.2)
Pleural fluid	2 (18.1)

IQR, Interquartile range.

^a^New images of infection appeared or old lesions worsened compared to previous chest radiographs.

The most frequent abnormal finding seen in chest x-ray was infiltration (n = 7, 63.6%). Cavitation, nodule, consolidation and pleural fluid came next in order of frequency. As for the thorax computed tomography (CT) findings, again infiltration was frequently encountered (n = 8, 72.7%). Nodule, consolidation, cavity and pleural fluid was next in order of frequency (Table 2). Progress in radiological findings for infection before antifungal treatment was detected in seven cases (63.6%).

### Antifungal treatment and results

Diagnostic procedures, treatment and results for all patients are shown in Table 3. In terms of treatment, all cases received amphotericin B [7 (63.6%) amphotericin B deoxycholate, 4 (36.4%) liposomal amphotericin B] for initial treatment and 5 (45.4%) patients were discharged with oral voriconazole. Six (54.5%) patients died before the completion of treatment and were considered as “treatment failure”. In 5 patients (45.4%), clinical and radiological improvement after antifungal treatment was followed up and these cases were acknowledged as “treatment success”. In those patients in whom treatment success was observed, the average duration of treatment was 137.2 days; instead it was 15.8 days in the patients who died.

**Table 3:** Diagnosis, antifungal therapy, and outcomes in 11 COPD patients with IPA

	**Diagnosis**	**Risk factor**^**a**^	**Chest x-ray**	**Thorax CT**	**Type of specimen**	**Isolation type**	**Antifungal treatment**	**GM value and control (ng/mL)**	**Beta D- Glucan (pg/mL)**	**Death**
1	Probable	Steroid	Infiltration, cavitation	Infiltration, cavity	Sputum, pleural fluid	*A. niger*	LAMB	3.79 → 0.24	none	Yes
2	Probable	Steroid	Infiltration	Infiltration	Bronchial lavage	*A. fumigatus*	LAMB	0.53 → none	none	Yes
3	Probable	Steroid	Cavitation	Cavity	Sputum	*Aspergillus species*	AMB	3.79 → 2.33	none	Yes
4	Probable	Steroid and antibiotic	Infiltration	Infiltration, consolidation	Sputum and bronchial lavage	*Aspergillus species*	AMB	9.16→ none	none	Yes
5	Probable	Steroid	Consolidation	Consolidation	Bronchial lavage	*A. niger*	AMB	5.48 → 4.38	none	Yes
6	Probable	Steroid	Cavitation, nodule	Infiltration, cavity, nodule	Sputum	*A. fumigatus*	AMB	0.22 → none	> 523	Yes
7	Proven^b^	Steroid and antibiotic	Consolidation, nodule	Consolidation, nodule	Sputum	*A. fumigatus*	AMB, then voriconazole	0.54 → 0.33	59	No
8	Proven^b^	Antibiotic	Infiltration	Infiltration, nodule	Bronchial lavage	*A. fumigatus*	AMB, then voriconazole	0.60 → 0.30	none	No
9	Probable	Steroid	Infiltration	Infiltration	Bronchial lavage	*A. fumigatus*	AMB, then voriconazole	0.50 → 0.16	51	No
10	Probable	Steroid	Infiltration, nodule	Infiltration, nodule	Sputum	*A. niger*	LAMB, then voriconazole	0.17 → none	90	No
11	Proven^b^	Steroid and antibiotic	Infiltration	Infiltration	Transbronchial biopsy	*A. fumigatus*	LAMB, then voriconazole	0.39 → none	> 523	No

^a^The term Steroid includes only systemic corticosteroid treatment.

^b^Hyphae were seen in the pathological examination of biopsy material.

AMB, Amphotericin B deoxycholate; IPA, Invasive pulmonary aspergillosis; LAMB, Liposomal amphotericin B.

## Discussion

In the present study, the diagnosis and treatment features of 11 COPD cases, who had IPA but no other immunosupressive diseases, were analyzed. In recent years, it has been shown that corticosteroid use plays a significant role in terms of increasing the rate of IPA incidence in COPD cases [5,6]. In one retrospective study, it was shown that steroid use of over 700 mg in total within the last three months in COPD patients increased the risk of IPA [6]. Also, it has been stated by some authors that inhaled steroids are a risk factor for IPA [14]. While 72.7% of cases in this study had used an inhaled steroid, 10 cases (90.9%) had used a systemic steroid within the last 3 weeks.

Another risk factor charged with the development of IPA in COPD patients is broad-spectrum antibiotic treatment. In one study, it was pointed out that treatment with a wide range of broad-spectrum antibiotics used before hospitalization in COPD cases was associated with the development of IPA and another study showed that using three or more antibiotics for 10 days was a risk factor for IPA development in patients with COPD [15,16]. In the present study, similar to other reports, four cases had used antibiotics in the last 3 months and a history of antibiotic use was the only risk factor in one case that did not receive systemic steroid treatment.

Since underlying disease may mask the findings of *Aspergillus* lung invasion, clinical symptoms and radiological findings are nonspecific in cases with COPD [6]. In COPD patients where IPA had developed, dyspnea was reported as the most frequent symptom and cough, wheezing, increase in sputum amount and fever may also be seen [16]. The most frequent radiologic finding was infiltration [6,7] that is non specific and may correspond to **a** COPD exacerbation caused by bacterial pulmonary infection, frequently encountered especially in chest disease clinics. For this reason, patients are frequently diagnosed as affected with IPA following their hospitalization. In our study, we were able to diagnose IPA in an average of 13.8 days after hospitalization. This also shows that an early screening test for IPA should be performed. In addition, the isolation of *Aspergillus* strains in culture and no regression in radiological and clinical findings with appropriate antibiotic treatment are the most important findings which suggest IPA. In this study, similar to other reports, the dyspnea was reported in all cases and the chest x-rays of seven cases at follow up showed a progression of findings compared to the chest x-ray on hospital admission.

In studies carried out on patients with hematologic malignancy from our country, **GM** antigen test sensitivity was found as 56-61% and its specificity as 21-84% [17,18]. However, in studies carried out on COPD cases in different centers, GM antigen test sensitivity in the diagnosis of IPA was stated as 46.2-80% and specificity as 83.3-94.1% [16,19,20]. In addition, it has been indicated that GM positivity in COPD patients with IPA development in intensive care and the isolation of *Aspergillus* species in respiratory samples may be significant in terms of mortality [3]. As for our study, the GM value was found to be positive in 9 (81.8%) cases, and 5 of them (55.5%) died. This gives further support to the belief that GM positivity in COPD cases with the development of IPA is a significant parameter to be considered in terms of mortality. Moreover, it is conceivable that a GM value positive in the diagnostic period will become negative in cases responding to treatment [21]. In 2 of our 7 cases where the GM test was repeated after they received antifungal treatment for at least one week, it was found that GM value was still positive, in fact treatment failed in both patients and they died (Table 3). While 1,3-beta-D-glucan was found to be negative in two cases for whom the GM value was positive, it was found positive in three IPA cases with negative GM test. In a study carried out recently, the sensitivity of the 1,3-beta-D-glucan test in the diagnosis of IPA was reported as 66% and its specificity as 75.6% [22]. Especially in patients from whom a bronchoscopic sample was not taken, applying both serologic methods may increase diagnostic sensitivity, although prospective systematic studies analyzing this application in patients with COPD are needed.

Even though amphotericin B has been used as the first-line antifungal agent in the treatment of IPA for many years, in a comparative study carried out in 2002 voriconazole was more successful than amphotericin B as the primary mode of invasive aspergillosis treatment [23]. Saito et al. also observed a good response as high as 60.6% with voriconazole in patients with chronic pulmonary aspergillosis [24]. In a recent study, Chu et al. reported 108 invasive fungal infections treated with voriconazole in whom 83 patients (76.8%) were IPA [25]. Although the authors, in patients with proven and probable invasive fungal disease, did not find a significant relationship between therapeutic voriconazole levels and achievement of complete or partial response at 12 weeks, radiologic response was noted in 55.2% of patients with invasive pulmonary aspergillosis. In addition, they found complete or partial response at 6 weeks with initial subtherapeuticvoriconazole levels in seven patients all affected with invasive pulmonary aspergillosis. In the present study partial response was achieved in 45.4% of patients and all received voriconazole after initial amphotericin B therapy. This was due to the fact that treatment in living cases was continued with the use of voriconazole by virtue of its oral form being available.

In the studies of different centers, the mortality rate of IPA in COPD cases was reported to be between 43.3% and 95% [5-7,16,26]. In our study, the mortality rate was 54.5%, however, to define the excess cause of mortality is difficult in this patient group since they had a very limited lung function based on the stage of COPD.

## Conclusions

In conclusion, we believe that “proven” or “probable” IPA should be taken into account in differential diagnosis, particularly in patients with severe and very severe COPD presenting with dyspnea exacerbation, poor clinical status, and a new pulmonary infiltrate on chest radiograph under treatment with broad-spectrum antibiotics and steroids. The presents study shows that the LRT culture for *Aspergillus*, serum GM test and chest imaging (x-ray and CT) can be useful for the early differential diagnosis of IPA, and voriconazole should be given for the treatment of such patients.

## Availability of supporting data

The data set supporting the results of this article is included within the article.

## Competing interests

The authors declare that they have no competing interests.
